# Evaluating the Effect of Hydrophobic Nanosilica on the Viscoelasticity Property of Asphalt and Asphalt Mixture

**DOI:** 10.3390/ma11112328

**Published:** 2018-11-19

**Authors:** Wei Guo, Xuedong Guo, Mengyuan Chang, Wenting Dai

**Affiliations:** 1 School of Transportation, Jilin University, Changchun 130022, China; guowei17@mails.jlu.edu.cn (W.G.); guoxd@jlu.edu.cn (X.G.); 2 China Communications Second Highway Survey Design and Research Institute Co. Ltd., Wuhan 4300704, China; chenwx16@mails.jlu.edu.cn

**Keywords:** viscoelasticity, nanomaterial, hydrophobic nanosilica, hydrophilic nanosilica

## Abstract

Viscoelasticity property of bitumen is closely related to the service life of bituminous pavement. This paper evaluated the impact of one of the most efficient and widely used nanomaterials in various industries called hydrophobic nanosilica on the viscoelasticity property of bitumen and asphalt mixture. In this paper, three hydrophobic nanosilica modified bitumens and asphalt mixtures were researched by conventional physical properties test, SEM test, FTIR test, DSC test, DSR test, static creep test and dynamic creep test. The results showed that the introduction of hydrophobic nanosilica could strengthen the viscosity of asphalt more effectively and had better dispersion than hydrophilic nanosilica in asphalt. From conventional physical properties test and rheological performance test, hydrophobic nanosilica could weaken the temperature susceptibility of bitumen observably. From DSR test, hydrophobic nanosilica modified asphalt had a lower sensitivity and dependence on temperature and frequency than hydrophilic nanosilica modified asphalt. The Cole–Cole diagrams indicated that hydrophobic nanosilica exhibited good compatibility with asphalt compared with hydrophilic nanosilica. Newly formed chemical bonds were found in the hydrophobic nanosilica modified asphalt and its mixture with stone according to SEM test, FTIR test, and DSC test, which is the biggest difference from the modification mechanism of hydrophilic nanosilica modified asphalt. Through static and dynamic creep test, it found that the addition of hydrophobic nanosilica can significantly reduce the creep strain at the same temperature.

## 1. Introduction

Asphalt material has been widely used in airfield and high-grade road pavement due to its numerous advantages such as smoothness, low-vibration, high-automated construction, and easy-maintenance [1,2,3]. As a typical viscoelastic material, the damage of asphalt pavement mainly depends on some external factors such as temperature, stress, oxygen, water and so forth. With the effect of temperature, vehicle, water, and oxygen, some damages including low-temperature cracking, high-temperature rutting, moisture-induced damage and deterioration of pavement structure would be generated easily [4]. He et al. investigated the rheological properties of lignosulfonate intercalated layered double hydroxides modified bitumen (LLMB) before and after UV aging by temperature susceptibility, dynamic viscoelastic properties and rheological model analyses. The results showed that the introduction of lignosulfonate intercalated layered double hydroxide (LS-LDHs) could strengthen the anti-UV aging ability of bitumen more effectively [5]. Liu et al characterized the effect of graphene oxide on viscosity, rheological properties, creep and recovery behavior, cracking resistance, and thermal properties of unaged/RTFO aged binders. The results demonstrated that the addition of trace amounts of graphene oxide (no more than 0.2 wt %) enhanced the paving temperature (120–135 °C) viscosity, high-temperature elasticity and rutting resistance of non-modified/SBS modified binders [6]. Gao et al. study the rheological behavior and the high-temperature performances of bio-asphalt binders. The results indicated the incorporation of bio-oil reduced the anti-rutting performance of asphalt [7]. In consequence, to lengthen the service life of the asphalt pavement, it is crucial to enhance overall performance and investigate rheological property of asphalt.

Because of the complexity of the pavement damage phenomenon, using proper additives and modifiers is considered the most cost-effective technique for prolong the pavement service life [8]. The modification of asphalt binder with polymeric materials and rubber powder is among the most common methods to improve the performance of the asphalt binder against most types of distresses [9,10,11]. The other method for prolonging the pavement service life is the addition of alternative environmental friendly materials. The use of industrial waste is a promising solution to minimize environmental pollution by sinking the accumulation of waste materials, which results in a reduction of the construction costs [12]. Golewski evaluated the effect of the low calcium fly ash (LCFA) addition on fracture processes in structural concretes. The results presented the properties of composites with the additive of LCFA depend on the age of the concrete tested, and mature concretes exhibit high fracture toughness at 20% additive of LCFA [13]. Woszuk analyzed the synthetic zeolites produced from fly ashes with 4 different types of crystalline structure. It was found that the viscosity of asphalt pastes with zeolitic materials increases with the increase in the amount of zeolite added. The potential usefulness of fly ash derived zeolites had also been proved [14]. Zhang et al. developed an innovative measure to solve this waste disposal problem by means of synthesizing zeolite A from the Sewage Sludge Ash (SSA) and using it as a Warm Mix Asphalt (WMA) additive, which can decrease the construction temperature of asphalt pavement, thus reducing the associated energy consumption and pollutant emission [15]. Another method for the improvement of asphalt binder characteristics that has recently gained attention is the modification of asphalt binder with nanomaterials [16]. Nano-material refers to a material with the size of less than 100 nmin at least one dimension. Due to the very tiny size and huge surface area, the properties of nanomaterials are much different from the normal-sized materials. The addition of these nano-sized particles to another material may overcome the limitations of monolithic, and asphalt binder is no exception [17]. Adding nanomaterials changes the rheological properties of the asphalt binder and might also lead to changes in the intermolecular forces within the asphalt binder structures. This is due to the fact that as dimensions reach the nanometer level, interactions at phase interfaces become largely increased. Nano silica is one of the nano-materials that have been extensively used in asphalt mixtures. Taherkhani et al. investigated the effects of nanosilica modification on some properties of a penetration grade asphalt cement and a typical asphalt concrete. The results showed that the addition of nanosilica results in the increase of stiffness, tensile strength, resilient modulus, fatigue life and resistance against permanent deformation and moisture damage [18]. Saltan et al. evaluated the performance of Hot Mix Asphalt (HMA) and bitumen by modifying bitumen with nanosilica according to Superpave mix design procedure. The results showed that nanosilica modified bitumen has the highest rutting performance, fatigue performance [19]. Firouzinia et al. examined the impact of nanosilica on the thermal properties of bitumen and asphalt mixture. The results showed that nanosilica can improve the temperature sensitivity of asphalt mixtures [20].

However, nanosilica as a kind of inorganic non-metallic nanomaterials is very prone to agglomeration. In addition, the asphalt binder is an organic cementitious material formed by high molecular hydrocarbons and non-metallic derivatives of these hydrocarbons, which makes nanosilica in asphalt binder have poor dispersibility and compatibility [21]. In order to improve the dispersion of nanosilica in organic solvents and enhance their interaction with the medium, and to broaden the application field of nanosilica, the commonly used method is to physically or chemically react the surface of the nanosilica with the surface modifier through a certain processing process [22]. The surface modification method can reduce the number of silanol structures on the surface of the nanosilica, change the structure of the surface functional groups and the atomic layer of the nanosilica particles. Thereby, the surface properties such as physicochemical adsorption of nanosilica are changed, the surface free energy of the nanoparticle and the phenomenon of agglomeration between particles is reduced.

The hydrophobic nanosilica obtained by modifying the surface of nanosilica has been applied in many fields, such as silicone rubber, thermosetting plastics, traditional coatings and many other product preparation [23,24]. Wang et al. incorporated hydrophobic nanosilica sol into polyvinylmethy-lsiloxane to prepare reinforced high-temperature vulcanized (HTV) silicone rubber. The resulted showed that HTV silicone rubber filled with 40 phr hydrophobic nanosilica sol has excellent mechanical and optical properties [25]. Ani et al. synthesized a series of nano-hybrid perfluoroalkyl oligosiloxane resins (FSi@SiO_2_) using the hydrolysis and condensation of FVPS with tetraethylorthosilicate to improve the hydro-and oleo-phobic properties of anti-fingerprint coating [26]. Milionis et al. synthesized acrylonitrile butadiene styrene rubber (ABS) superhydrophobic nanocomposites containing various concentrations of hydrophobic silica nanoparticles by solution processing and spray coating on aluminum surfaces. The resulted showed that superhydrophobic nanocomposites has excellent wear abrasion resistance [27].

To the authors’ knowledge, the open literature has no experimental studies of evaluating the effect of hydrophobic nanosilica on the viscoelasticity property of asphalt and asphalt mixture. In this study, the viscoelasticity of three hydrophobic nanosilica modified bitumens and asphalt mxitures were researched by laboratory test. Conventional physical properties test was used to prove the availability of hydrophobic nanosilica to bitumens.The SEM test, FTIR test, DSC test were carried to explore the modification mechanism of hydrophobic nanosilica. Moreover, the DSR test and creep test was employed to systematically investigated the effect of hydrophobic nanosilica on the viscoelasticity property of asphalt and asphalt mixture.

## 2. Materials and Sample Preparation

### 2.1. Asphalt

The asphalt used in this study was Donghai 70# asphalt produced by Qilu Branch of Sinopec Corp. (Qingdao, China). Laboratory tests were carried out in order to assess the conventional properties of asphalt according to Chinese standards. The test results of the asphalt used in this study are summarized in Table 1.

### 2.2. Hydrophobic Nanosilica

**Figure 1:**
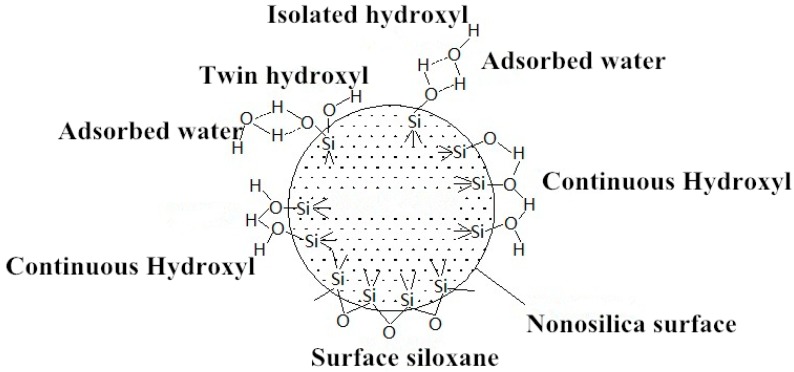
Surface structure of nanosilica.

The molecular formula of nanosilica is SiO_2_·H_2_O, which is an amorphous structure of SiO_2_. The surface structure of nanosilica is shown in Figure 1. The hydroxyl groups on the surface can be classified into three types: isolated hydroxyl group, continuous hydroxyl group, and twin hydroxyl group. Isolated hydroxyl group is a free hydroxyl group that does not participate in the reaction; continuous hydroxyl group is formed by two hydrogen groups that generate hydrogen bonds and associate with each other; two hydroxyl groups attached to the same Si are called twin hydroxyl groups. Isolated hydroxyl group and continuous hydroxyl group have no hydrogen bond. The reinforcing effect of nanosilica as a modifier mainly comes from the active structure (–Si–OH) on the surface of the particles, and it is easy to bond the surrounding ions. The surface of nanosilica is uniformly distributed with a layer of silanol and siloxane group with strong water absorption. The water molecules can be physically covered on the surface of the particles or chemically bonded to the hydroxyl groups on the Si atoms. Therefore, nanosilica shows a strong affinity for water. In order to reduce the number of surface silanol structures and change the surface functional groups of the primary nanosilica particles, the surface modifier is used to modify the nanosilica to realize the transition from hydrophilic to hydrophobic.

The hydrophobic nanosilica used in this research included organochlorosilane surface modified nanosilica (OCS-Silica), silicone organic compound surface modified nanosilica (SOC-Silica) and silane coupling agent surface modified nanosilica (SCA-Silica). The nanosilica and three hydrophobic nanosilica materials are obtained from Changtai Weina Chemical Co., Ltd. (Shouguang, China). The technical properties of four nanosilica materials provided by Changtai Weina Chemical Co., Ltd. (Shouguang, China) have been presented in Table 2.

### 2.3. Preparation of Samples

According to prior researches, mixing of nanoparticles into asphalt binder with a high shear mixer at the rate of more than 4000 RPM, at a temperature between 130 °C and 165 °C, and an approximate duration of 40 min to an hour is suggested [28]. It should be noted that the mentioned studies used various types of nanoparticles including surface modified ones which might need less mixing speed or mixing time to achieve proper dispersion.

Based on the modified asphalt preparation test, a mixture of nano Silica, nano OCS-Silica, nano SOC-Silica and nano SCA-Silica with asphalt binder is produced. 3 wt % nano Silica, nano OCS-Silica, nano SOC-Silica or nano SCA-Silica and the base asphalt were taken into a pot heated the mixture to 140 °C with oil-bath temperature control system. Then the mixture was stirred with the speed of 2000 rpm for 1 h to ensure homogeneous blending, to obtain nano Silica modified asphalt, OCS-Silica modified asphalt, SOC-Silica modified asphalt or SCA-Silica modified asphalt. The asphalt modified by nano Silica nano OCS-Silica, nano SOC-Silica and nano SCA-Silica were denoted by SBA, OCSSBA, SOCSBA, and SCASBA, respectively. Meanwhile, base asphalt was denoted by BA.

## 3. Experimental Procedures

### 3.1. Conventional Physical Properties Test

The conventional physical properties tests on BA, SBA, OCSSBA, SOCSBA, and SCASBA Such as softening point, ductility and penetration were performed according to Chinese standards GB/T0606-2011, GB/T0605-2011, and GB/T0604-2011, respectively. The viscosity test was carried out using a Brookfield rotational viscometer to measure the apparent viscosities of modified and unmodified asphalt binders according to Chinese standards GB/T0625-2011. In order to evaluate the aging resistance property of asphalt, the rolling thin film oven (RTFO) test was conducted to simulate the effect of construction aging according to Chinese standards GB/T0610-2011. After the rolling thin film oven tests, damaged specimens were collected for ductility test, penetration test and weighing test to identify the aging resistance property of modified asphalt.

### 3.2. SEM Test

SEM has been used to investigate the micromorphology of asphalt binders and mixtures. These microstructure further investigated into fracture morphology, combustion properties, rheological properties, modification mechanism interaction between modifier and aggregate, structural, viscoelastic and physicochemical properties. The microstructure of three hydrophobic nanosilica materials and nanosilica were examined by SU8000 electronic microscopy (Tianmei Co., Tokyo, Japan). The hydrophilic nanosilica and hydrophobic nanosilica particles were dispersed onto double-sided adhesive carbon tape, which was applied to the aluminum sample mounts. For the operation of SU8000 electronic microscopy, the accelerating voltage was 1–5 kV, the emission current was 9 A, and the working distance was 2.8–8 mm. 

### 3.3. FTIR Test

Different molecular structures and functional groups have diverse absorptions of infrared radiation. Therefore the chemical composition can be identified by analyzing the infrared absorption spectra of samples. A Vertex 70 Fourier Transform Infrared Spectroscope (BRUKER OPTICS, Changchun, China) was used for the determination of the chemical compositions of both neat and modified binders. Spectrometry was performed at wavenumbers between 400 and 4000 cm^−1^ with a wavelength wavelength accuracy of 0.01 cm^−1^, resolution of 4 cm^−1^, the rate of 0.16 cm/s, and 32 scans. A small amount of each sample was placed on a diamond ATR prism and a certain amount of pressure was applied for all the samples.

### 3.4. DSC Test

Differential scanning calorimetry (DSC) analyzes energy differences between the neat and modified asphalt binders at different temperatures. DSC tests were conducted using a DSC-500B (Shanghai yinuo precision instrument Co. Ltd., Changchun, China) to get the thermographs of both neat and modified asphalt binders. Approximately 5 mg of each asphalt sample was used in the test. The range of the testing temperature was from −60 °C to 50 °C, and the heating rate was 5 °C/min under a nitrogen atmosphere. Two parameters, glass transition temperature *T_g_* and endothermic energy value *Δcp*, were used to quantify the thermal properties of both neat and modified asphalt binders. Three specimens of each asphalt were measured to ensure reliability of the DSC results [29,30].

### 3.5. DSR Test

The viscosity and elasticity of asphalt binders can be evaluated using the DSR machine based on parameters such as complex modulus (G*), phase angle (δ), Shenoy rutting parameter (G*/[1-(sinδtanδ)^−1^]), storage modulus (G*·cosδ).

In this paper, temperature sweep test and frequency sweep test were performed using a Bohlin automatic dynamic shear (ADS) rheometer (DSRII, Malvern, UK), including the host, rheometer measuring system or measuring fixture, temperature control unit, and the instrument software.

#### 3.5.1. Temperature Sweep Test

Temperature sweep tests were conducted on modified and unmodified asphalt binders samples under control deformation mode at strain equals to 12% and frequency equals to 1.59 Hz. This frequency of oscillation can simulate the shear stress corresponding to a traffic speed of approximately 100 Km/h. Complex shear modulus (G*) and phase angle (δ) were measured at temperatures ranging from 28 °C to 76 °C at 2 °C increments for both asphalt binders. The diameter of the asphalt sample fixture was 25 mm, while the test spacing of asphalt sample was 1 mm.

#### 3.5.2. Frequency Sweep Test

The frequency sweep tests were carried out to evaluate the shear-deformation performance and temperature sensitivity of the hydrophobic nanosilica modified binders. The frequency ranged from 0.1–200 rad/s and these tests were conducted at four different temperatures (i.e., 22 °C, 40 °C, 58 °C, and 76 °C). The parallel plates with diameter of 25 mm and gap of 1 mm were selected for the tests at the medium-high temperature range (e.g., 58 °C, 76 °C), whereas parallel plates with diameter of 8mm and gap of 2 mm were selected for the tests at the low-medium temperature range (e.g., 22 °C, and 40 °C).

From all of the two tests, the linear viscoelastic parameters such as G* (complex shear modulus) and δ(phase angle) was obtained. Meanwhile, the Shenoy rutting parameter G*/[1−(sinδtanδ)^−1^] and storage modulus G*·cosδ was calculated. The G* represents the binders’ ability of resistance to deformation and the δ reflects the binders’ viscoelastic ratio. Consists of G* and δ, the Shenoy rutting parameter G*/[1−(sinδtanδ)^−1^] is adopted as the index representing the asphalt binders’ property (i.e., resistance to permanent deformation) and the storage modulus G*·cosδ is adopted as the index representing the asphalt binders’ elastic property [31].

#### 3.5.3. Master Curve Generation Method

The master curve was used to describle the rheological properties of hydrophobic nanosilica modified asphalt in this study. The master curve of G* was generated by the applying of the time-temperature superposition principle. To construct master curve, a reference temperature is selected and the dates collected from frequency sweeps at all other temperatures are shifted to one at reference temperature by shift factors. In this research, reference temperature was 40 °C.

#### 3.5.4. Compatibility Analysis Method

The modifiers of nanomaterial can modify the asphalt from the microstructure, which is fundamentally different from other conventional modifiers, and whether the nanoparticles can be uniformly dispersed microscopically, and formed a stable structure with the asphalt component is the key to improve the asphalt. Cole–Cole plots were the most effective methods to identify compatibility and these plots have been used widely to study compatibility in polyblends. As a consequence, Cole–Cole diagrams are employed to evaluate the compatibility of hydrophobic nanosilica modified asphalt. Cole–Cole diagrams consist of representations of the complex viscosity components (η* = η’ − η”) in the complex plane (η’, η”). Evolution of η” with η’ in conformity with symmetrical parabolas is considered as the proof of compatibility, while deviation from this symmetry is related to incompatibility for modified asphalt.

### 3.6. Viscoelastic Test of Asphalt Mixture

The viscoelasticity of modified asphalt mixtures can be evaluated by creep tests. SBA and SCASBA were selected to prepare asphalt concrete sample. The gradation of asphalt concrete is listed in Table 3. The Marshall mix design method is still widely used in the road laboratory, all around the world [32,33,34]. Optimal asphalt content of SBA-AC and SCASBA-AC mixture were ensured as 5.5% and 5.2% of dry aggregate using Marshall mix design method according to JTG E20-2011. The method of load application for creep testing in a laboratory environment can be classified into two types: static and dynamic loading. For both tests, the specimens were prepared according to Chinese standards GB/T0738-2011, with a diameter of 100 mm and height of approximately 63.5 mm. The specimens were kept in a climatic cabinet for 24 h.

#### 3.6.1. Static Creep Test

The test was performed as uniaxial static creep test, the specimen was subjected to a constant compressive load of 150 kPa at a test temperature of 30 °C, 40 °C, 50 °C, 60 °C. In this work, the test was performed for 3600 s.

#### 3.6.2. Dynamic Creep Test

In a dynamic creep test, a repeated uniaxial stress is applied to an asphalt specimen for a number of load cycles while axial strain is measured in the same direction as the loading using linear variable differential transducers (LVDTs). The applied dynamic load used in this test was a sequence of rectangular pulses. The pulse duration was 1 s, and the rest period before the next pulse was 1 s. The test was repeated 1800 times. A static axial stress of 15 KPa was applied for 90 s to the top platen of the specimen for proper bedding, as in a static creep test. The deviator stress repeated loading was 150 kPa, and the testing temperature was set to 30 °C, 40 °C, 50 °C, and 60 °C. 

## 4. Results and Discussion

### 4.1. Conventional Physical Properties Analysis

The results of empirical tests such as penetration, softening point, and 10 °C ductility for unmodified and modified asphalt are given in Table 4. It is readily seen by reference to this Table that the physical properties of asphalt were greatly affected following the addition of modifier. From penetration test, it was observed that addition of three hydrophobic nanosilica materials reduced the temperature sensibility of asphalt, but hydrophilic nanosilica is the opposite. The reason for the improvement in PI values of three hydrophobic nanosilica materials modified binder samples is the hydrophilic nanosilica is an inorganic modifier and is prone to agglomeration. Segregation and uneven dispersion may occur in the modified asphalt material, and the improvement of the temperature sensibility of the asphalt is not obvious. The hydrophobic nanosilica after surface modification is more compatible with asphalt, which is beneficial for adsorbing light oil (OCS-Silica, SOC-Silica) in the asphalt or bonding with some components in the asphalt (SCA-Silica), the original structural form of the asphalt is changed, so that the temperature sensitivity of the asphalt after the modification is lowered. From softening point test, it was observed that addition of hydrophobic nanosilica and hydrophilic nanosilica could improve the softening point. From 10 °C ductility test, it was observed that the ductility of modified asphalt binders was very close, and the ductility of hydrophobic nanosilica modified asphalt is slightly lower than that of hydrophilic nanosilica modified asphalt. Based on conventional tests, it can be concluded that addition of hydrophobic nanosilica results in relatively harder binder compared to SBA, which may be beneficial for rutting resistance.

The high-temperature viscosity of asphalt was used to evaluate if the asphalt is fluid enough during pumping and mixing. Table 5 shows the viscosity of five asphalt binders at 115 °C, 135 °C and 155 °C. The viscosity of control BA binder was found to be 0.4503 pa.s at 135 °C, whereas SBA, OCSSBA, SOCSBA, and SCASBA showed viscosity of 0.7724 pa.s, 1.125 pa.s, 2.978 pa.s, and 1.987 pa.s. From the result it can be observed that all the binder combinations satisfied the Superpave specification (<3 pa.s). This shows that the satisfactory workability of asphalt mixes can be achieved by blending OCSSCA, SOCSBA, and SCASBA. The sequence of viscosity for the modified and unmodified asphalt binder were SOCSBA > SCASBA > OCSSBA > SBA > BA, respectively. This indicates that the addition of OCS-Silica, SOC-Silica, and SCA-Silica obviously increased the viscosity of asphalt compared with nanosilica, and SOC-Silica can improve the viscosity of asphalt most effectively among the four modifiers.

The paper used the Saal formula recommended by ASTMD 2493 to analyze the viscosity test results of asphalt binder samples. The Saal formula is as follows.

(1) $$lglg\left(\eta \times 10^{3}\right)=n-m\times lg\left(T+273.13\right),$$

Where η is the viscosity, T is the temperature, and the n, m is the regression coefficient. The regression coefficient *m* represents the temperature sensibility of asphalt. 

The *m* value of BA, SBA, OCSSBA, SOCSBA, and SCASBA was −3.234, −3.253, −2.697, −1.906, and −2.252. It can be seen that the addition of three hydrophobic nanosilica materials reduced the temperature sensibility of asphalt obviously compared nanosilica. This conclusion is consistent with the results of penetration test.

The RTFO test was conducted for five asphalt binders. In the standard RTFO test, the binders were conditioned at 163 °C, for 85 min, with sufficient air. Then, the RTFO-aged samples were used for penetration test, 10 °C ductility test and weighing test. Table 6 shows the test results of five asphalt binders after RTFO test. The mass change of asphalt during RTFO tets is usually due to the volatilization of light oil and the oxygen gain in chemical reaction, and the volatile weight loss is much larger than oxygen gain. Thus, the mass loss rate can reflect the anti-aging ability of asphalt, and the smaller the mass loss rate, the better the anti-aging performance. After calculation, it is found that both hydrophilic nanosilca and hydrophobic nanosilca can effectively reduce the mass loss of BA after aging, but the mass loss of hydrophobic nanosilica modified asphalt is relatively smaller. The main reason may be that the hydrophobic nanosilica after surface modification has stronger lipophilicity and can adsorb more light oil in the asphalt. In the process of thermal oxygen aging, the hydrophobic nanosilica better reduces the volatilization of the light oil than the hydrophilic nanosilica, thereby improving its anti-aging property.

The structural composition of asphalt after aging changes to show hardening. The 25 °C residual penetration ratio is the percentage of penetration at 25 °C of asphalt before and after aging. The greater the 25 °C residual penetration ratio, the less the degree of hardening of the asphalt, and the better the anti-aging ability. The 25 °C residual penetration ratio of five asphalt binders was 63.2%, 68%, 74.1%, 67.4%, and 69.1% respectively. This indicated that hydrophobic nanosilca is added into asphalt to form a new structure that can resist thermo-oxidative ageing and relieve asphalt hardening.

The 10 °C residual ductility is the percentage of ductility at 10 °C of asphalt before and after aging. The 10 °C residual ductility can reflect the low temperature performance of asphalt binders after RTFO test. The larger 10 °C residual ductility is, the better the anti-aging ability of asphalt. After RTFO, the ductility of BA decreased from 45.7 cm to 12.2 cm, and the 10 °C residual ductility of BA is 26.7%. The 10 °C residual ductility of SBA, OCSSBA, SOCSBA, and CASBA was 42.8%, 58.4%, 66.5% and 50.6%. This indicated hydrophobic nanosilica can effectively improve the low temperature mechanical properties of asphalt after aging compared with hydrophilic nanosilica.

### 4.2. SEM Analysis

**Figure 2:**
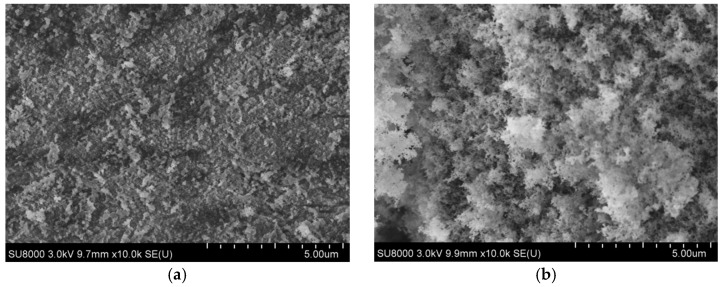

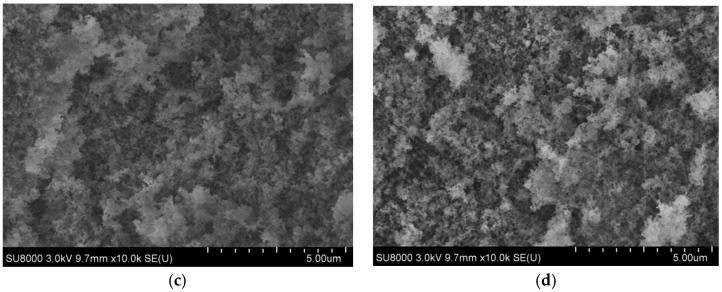
SEM images of nanosilica and three hydrophobic nanosilica materials at magnifications of ×10,000. (**a**) Silica; (**b**) OCS-Silica; (**c**) SOC-Silica; (**d**) SCA-Silica.

**Figure 3:**
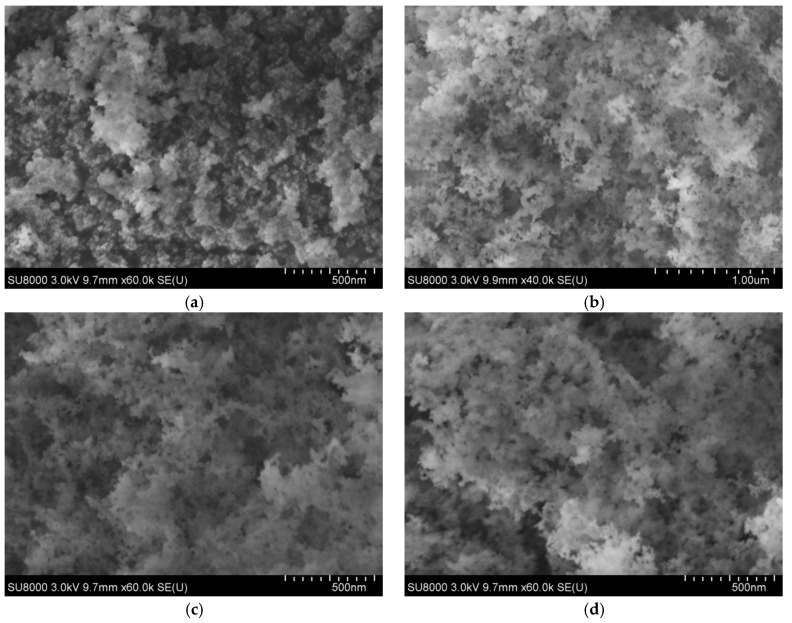
SEM images of nanosilica and three hydrophobic nanosilica materials at magnifications of ×60,000. (**a**) Silica; (**b**) OCS-Silica; (**c**) SOC-Silica; (**d**) SCA-Silica.

The microstructure of three hydrophobic nanosilica materials and nanosilica were examined by SU8000 electronic microscopy (Tianmei Co., Tokyo, Japan). The scanning electron micrographs of nanosilica and three hydrophobic nanosilica materials observed at magnifying power of ×10,000 and ×60,000 are shown in Figure 2a–d and Figure 3a–d.

It can be seen from Figure 2 that the primary SiO_2_ particles in the unmodified nannosilica are a zero-dimensional spherical nanomaterial, and the particles of the three hydrophobic nanosilica materials have a 3D continuous structure composed of many nanoparticles. These characteristics are highly valuable for hydrophobic nanosilica applications, serving excellent steric stabilization and ensuring efficient dispersion and good catalytic sites accessibility Further magnified SEM image of the four nanomaterials presented in Figure 3 indicates that a densely structured aggregate, agglomerate chain and a network structure formed by van der Waals force between spherical nanosilica particles in the hydrophilic silica, the structure can be easily destroyed by mechanical force, but it is easier to regenerate. The morphology of three hydrophobic nanosilica materials (OCS-Silica, SOC-Silica and SCA-Silica) is similar after surface modification. The main difference between the three hydrophobic nanosilica materials and the hydrophilic silica material is that the surface is grafted with organic groups. The high polymer forms an organic film on the surface of the nanosilica particles, and the organic film reduces the surface tension of particles,improves the agglomeration between particles and increases the dispersion in asphalt.

### 4.3. FTIR Analysis

**Figure 4:**
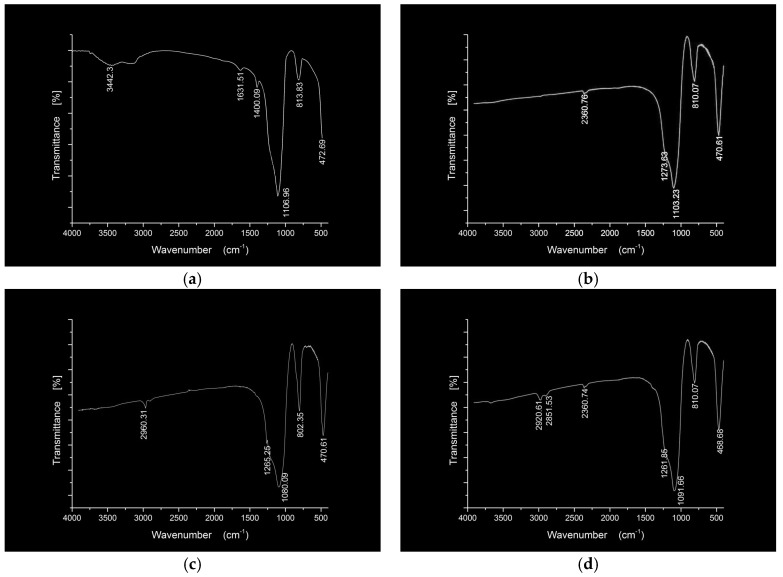
FTIR spectra of the nano silica and three three hydrophobic nanosilica materials. (**a**) Silica; (**b**) OCS-Silica; (**c**) SOC-Silica; (**d**) SCA-Silica.

The FTIR spectra of the nano silica and three hydrophobic nanosilica materials are given in Figure 4. Compared to the nanosilica, the FTIR spectra of three hydrophobic nanosilica materials has no absorption peak and wide absorption band in the region of 3700–3150 cm^−1^, indicating that most of the hydroxyl groups on the surface of nanosilica have chemically reacted with the modifier, resulting in the change of the surface properties of nanosilica. The OCS-Silica, SOC-Silica and SCA-Silica produced –CH_3_ symmetric deformation vibration peaks at 1273.63 cm^−1^, 1265.25 cm^−1^, and 1261.85 cm^−1^ respectively, indicating the organic structure of the modifier is grafted to the nanosilca. The new peaks around 2960.31 cm^−1^ corresponding to –CH_3_ and –CH_2_– stretching vibration from silicone modifier appeared in the FTIR spectra of the OCS-Silica, manifesting that the long chain of silicone organic compounds has been successfully grafted onto the surface of nanosilica. As can be seen from Figure 4d, the new peaks at 2920.61 cm^−1^ and 2851.53 cm^−1^ correspond to –CH_3_ and =CH_2_ antisymmetric stretching vibration from silane coupling agent. The observation suggested that the hydroxyl group on the surface of nanosilica reacted with the silane coupling agent, so that the nanosilica changed from hydrophilic to hydrophobic and lipophilic.

**Figure 5:**
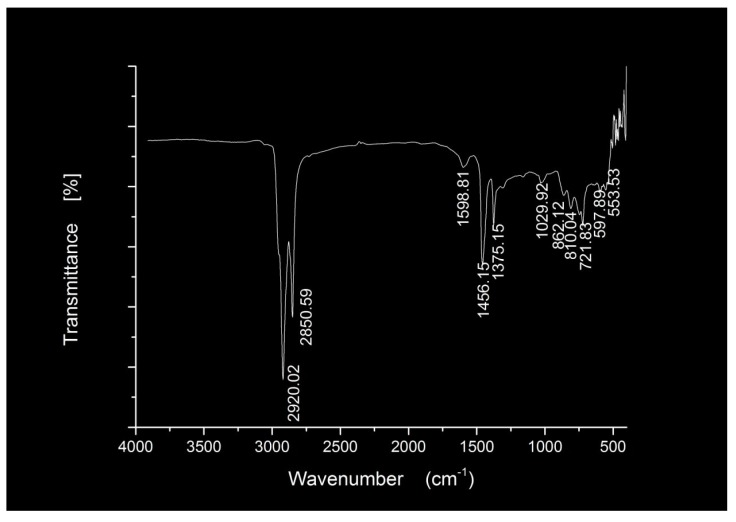
FTIR spectra of the BA materials.

**Figure 6:**
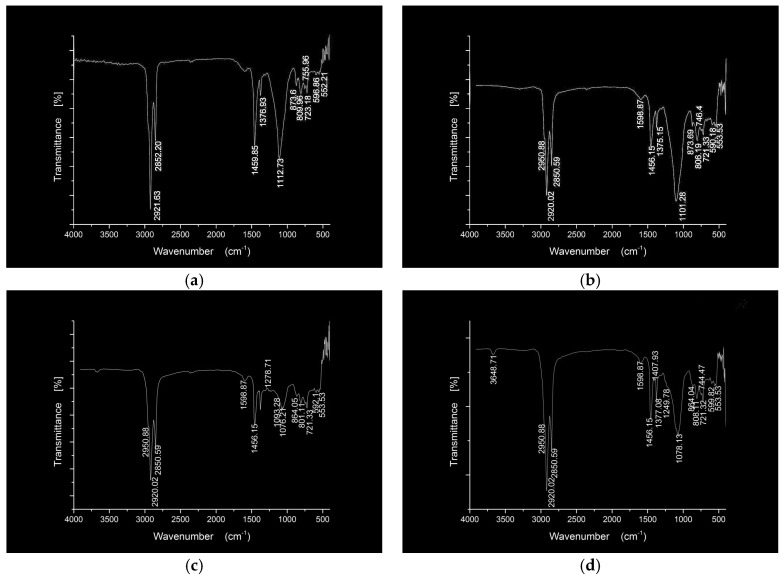
FTIR spectra of the SBA, OCSSBA, SOCSBA and SCASBA materials. (**a**) SBA; (**b**) OCSSBA; (**c**) SOCSBA; (**d**) SCASBA.

In order to further explore the interaction between hydrophobic nanosilica materials and asphalt binder, five asphalt binders were tested by infrared spectroscopy. The FTIR spectra of the neat and modified asphalt binders are given in Figure 5 and Figure 6.

As shown in Figure 5, two peaks emerged at 2920.02 cm^−1^ and 2850.59 cm^−1^, which correspond to –CH_2_– antisymmetric stretching vibration and symmetrical stretching vibration, suggesting the presence of a certain saturated hydrocarbon in the BA sample. The peaks at 1598.80 cm^−1^ correspond to C=C stretching vibration from benzene ring skeleton in aromatic. The peaks at 1456.15 cm^−1^ and 1375.15 cm^-1^ correspond to –CH_2_– flexural vibration and –CH_3_ symmetric deformation vibration (Umbrella vibration) from aliphatic series, respectively. The peak at 1029.92 cm^−1^ are attributed to S=O vibration, which indicates that the thermo-oxidative aging occurred between base asphalt during production and transportation. The peaks at 862.12 cm^−1^ and 810.04 cm^−1^ correspond to =C–H out-of-plane deforming vibration from polycyclic aromatic hydrocarbon. The peak at 721.83 cm^−1^ are attributed to alkyl radical flexural vibration. The observations illustrate that asphalt is a complex mixture of aliphatic compounds, saturated hydrocarbons, aromatic hydrocarbons and some heteroatom derivatives.

Compared to BA, the FTIR spectra of SBA is similar to BA. Only one obvious difference is that SBA produced Si–O–Si symmetrical stretching vibration at 1112.73 cm^−1^ from nanosilica, indicating the nanosilica does not chemically react with the asphalt,but a simple physical blending in the nanosilica modified asphalt. Compared to BA, three hydrophobic nanosilica modified asphalt produced a new absorption peak at 2950.88 cm^−1^ which is due to –O–Si(CH_3_) structure from OCS-Silica, the captain chain from SOC-Silica and methyl antisymmetric stretching vibration from SCA-Silica. Compared to BA, the SOCSBA produced O-Si stretching vibration at 1093.28 cm^−1^, and the peaks at 864.05 cm^−1^ and 801.11 cm^−1^ is heightened due to the superposition of Si–(CH_3_)_2_ stretching vibration from the captain chain. Compared to BA, the FTIR spectrum of SCASBA produced continuous peaks at 1407 cm^−1^ and 1249.78 cm^−1^. This may be due to the fact that the Q group on the surface of SCA-Silica reacts with the aromatic component in the modified asphalt, creating a new chemical bond. From the position of the peak, it can be inferred that the continuous peaks at 1407 cm^−1^ and 1249.78 cm^−1^ correspond to hydrogen bond stretching vibration from aromatic or aliphatic ether. Based on the above observations and analysis, it can be summarized that in addition to the physical blending reaction, there are certain chemical reactions in the three hydrophobic nanosilica modified asphalt.

**Figure 7:**
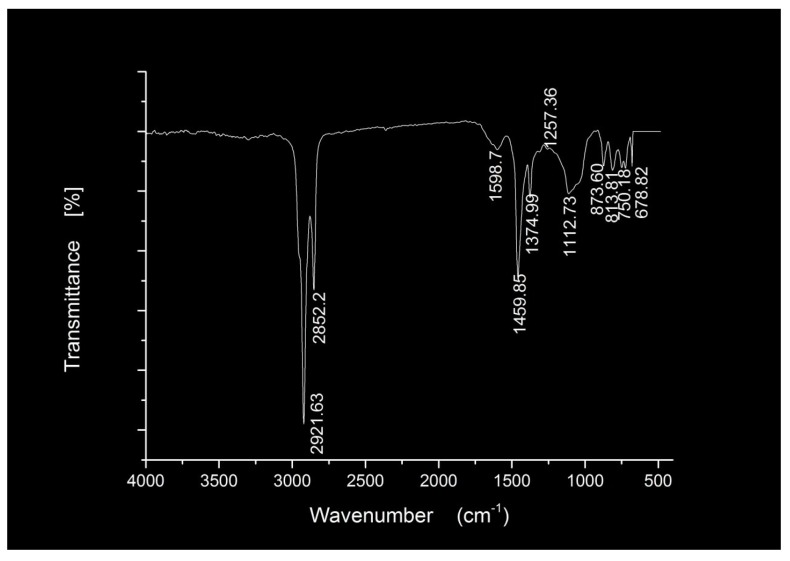
FTIR spectra of the BA-SP.

**Figure 8:**
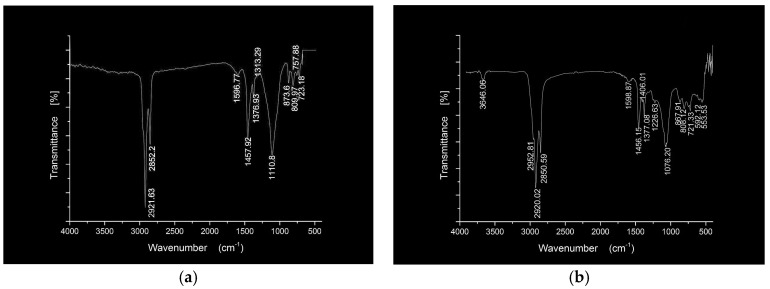
FTIR spectra of the SBA-SP and SCASBA-SP samples. (**a**) SBA-SP; (**b**) SCASBA-SP.

The early diseases occurrence of asphalt pavement, such as low-temperature cracking, high-temperature rutting, moisture-induced damage and so forth, has a great relationship with the adhesion between asphalt and aggregates. This adhesion is an extremely complicated physical-chemical process. In order to clarify the connection between the hydrophobic nanosilica modified asphalt and the stone, base asphalt mortar, nanosilica modified asphalt mortar and SCA-Silica modified asphalt mortar mixed with limestone power were tested by infrared spectroscopy. Limestone is an alkaline stone with an alkaline active center on the surface. It is easy to react with acidic components in asphalt to form water-insoluble compounds. Therefore, limestone is selected as the test material. The base asphalt mortar, nanosilica modified asphalt mortar and SCA-Silica modified asphalt mortar mixed with 5 wt % limestone power with a particle size of less than 0.15 mm were denoted by BA-SP, SBA-SP and SCASBA-SP, respectively. The FTIR spectra of the neat and modified asphalt mortar are given in Figure 7 and Figure 8.

Compared to base asphalt (Figure 5), the FTIR spectra of BA-SP is similar to BA. Only one obvious difference is that BA-SP produced a new peak at 1112.73 cm^−1^ from CaCO_3_, SiO_2_ and AI_2_O_3_ of limestone. Qualitative assessment of spectrums of BA-SP and SBA-SP show no significant peak except for wavenumbers at 1110.8 cm^−1^ for SBA-SP. The marked peak at 1110.8 cm^−1^ for this sample is related to the superposition of Si-O-Si from nanosilica and limestone power vibration and indicates no obvious chemical reaction in the SBA-SP. Qualitative assessment of spectrums of SBA-SP and SCASBA-SP show no significant peak except for wavenumbers from 1600–1200 cm^−1^ for the SCASBA-SP. The new continuous tiny absorption peak indicates some chemical bonds between nano SCA-Silica modified asphalt and limestone powder were formed, but the number was less.

### 4.4. DSC Analysis

**Figure 9:**
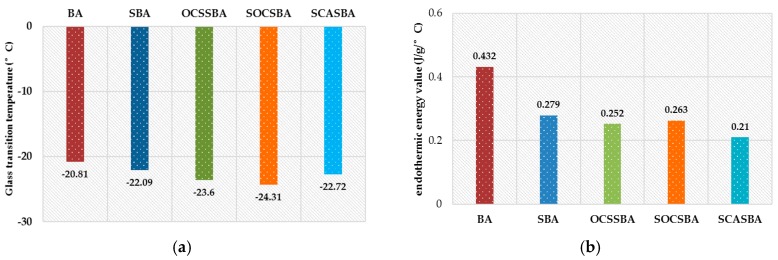
Differential scanning calorimetry (DSC) results of the neat and modified asphalt binders. (**a**) Glass transition temperature of the neat and modified asphalt binders; (**b**) Endothermic energy value of the neat and modified asphalt binders.

DSC was employed to evaluate the effect of nano silica and three hydrophobic nanosilica materials on the low-temperature thermal properties of the HMAs. Figure 9 presents the thermal properties of each type of studied asphalt binder, quantified by the glass transition temperature (*T_g_*) and endothermic energy value (*Δcp*). The value of *T_g_* indicates the temperature below which the asphalt binder changes from viscoelastic state to glassy state. The lower the *T_g_*, the better the asphalt to resist low-temperature cracking. The value of *Δcp* represents the difference in heat capacity before and after the glass transition of asphalt binder, which reflects the amount of energy required to change the collective state of the unit mass of asphalt binder. Under the same system, the lower the *Δcp* value, the higher the crosslinking density of the components in the asphalt binder. 

The incorporation of hydrophobic nanosilica materials greatly enhanced the low-temperature thermal properties of asphalt binder, as shown in Figure 9. The presence of 3 wt % nanosilica only resulted in a *T_g_* 1.28 °C lower than the base asphalt without the nanosilica. In contrast, the presence of 3 wt % hydrophobic nanosilica (OCS, SOC and SCA) resulted in a *T_g_* 2.79 °C, 3.5 °C 1.91 °C lower than the neat asphalt, respectively. This suggests that hydrophobic nanosilica is much more effective than nanosilica in reducing the glass transition temperature of the control asphalt. The addition of 3 wt % nanosilica in base asphalt decreased its *Δcp* value by 35.4%, and the presence of 3 wt % hydrophobic nanosilica (OCS, SOC and SCA) decreased the *Δcp* value of the base asphalt by 41.7%, 39.1% and 51.4%, respectively. These results demonstrate that the hydrophobic nanosilica improved the intermolecular crosslinking density in asphalt binders. According to the above FTIR spectra analysis, the hydrophobic nanosilica can chemically react with the control asphalt and thus restrict the movements of asphalt molecules, which may explain the observed increases in the crosslinking density of the asphalt binder.

In summary, adding hydrophobic nanosilica into asphalt will reduce the glass transition temperature of the binder (especially SOC modifier), and it will increase the crosslinking degree of the asphalt.

### 4.5. DSR Analysis

#### 4.5.1. Temperature Sweep Test Results

The temperature sweep tests of BA, SBA, OCSSBA, SOCSBA, and SCASBA were carried out, and the complex shear modulus (G*), phase angle (δ), Shenoy rutting parameter G*/[1−(sinδtanδ)^−1^] and black diagram were analyzed.

**Figure 10:**
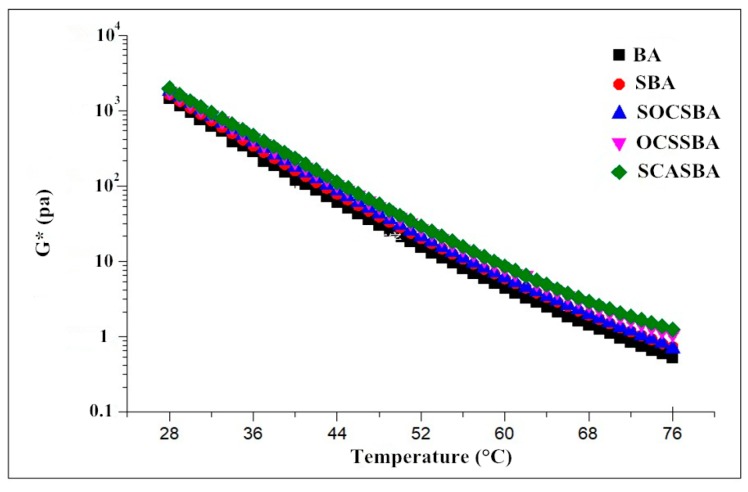
The relationship between complex shear modulus and temperature for modified and unmodified asphalt.

As seen in Figure 10, for BA, SBA, OCSSBA, SOCSBA, and SCASBA, the complex shear modulus decreased with the increase of temperature,this indicated that, with the increase of temperature, the asphalt became soft, and its resistance to rutting declined. For the temperature range of 28–46 °C, the complex shear modulus of modified and unmodified asphalt binder is similar, the sequence of complex modulus for the modified and unmodified asphalt binder were G* (SCASBA) > G* (OCSSBA) > G* (SOCSBA) > G* (SBA) > G* (BA), respectively. The results showed that the addition of nanosilica and hydrophobic nanosilica provided a certain rheological resistance of asphalt. Moreover, the complex modulus of hydrophobic nanosilica modified asphalt was always greater than nanosilica modified asphalt, which is because the hydrophobic nanosilica particles have higher affinity with asphalt binder functional groups through surface attraction. For the temperature range of 46–76 °C, the complex shear modulus of five asphalt binders has a gap. The complex shear modulus of SOCSBA decreases faster than SBA after 68 °C, but still higher than BA. The OCSSBA and SCASBA have higher complex shear modulus, indicating that OCS-Silica or SCA-Silica can effectively improve the strength of asphalt and its ability to resist deformation. Moreover, a higher slope of the fitted line means that a higher temperature sensitivity of the asphalt is observed. As can be witnessed, the temperature sensitivity of hydrophobic nanosilica modified asphalt was lower than that of nanosilica modified asphalt and base asphalt, which is consistent with the conclusions of the conventional physical properties test.

**Figure 11:**
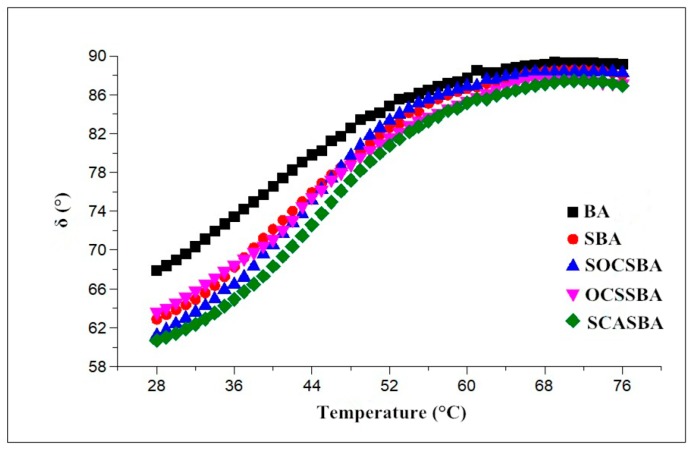
The relationship between complex shear modulus and temperature for modified and unmodified asphalt.

As shown in Figure 11, for BA, SBA, OCSSBA, SOCSBA, and SCASBA, the phase angle gradually increased with the increase of temperature, this indicated that the viscous component increased with increasing temperature. The sequence of initial phase angle for the modified and unmodified asphalt binder were δ (SCASBA) > δ (SOCSBA)> δ (SBA) > δ (OCSSBA)> δ (BA), respectively. This indicated that the elastic components of modified asphalt increased with the addition of the nanosilica and hydrophobic nanosilica. For the temperature range of 52–76 °C, the increase of the phase angle of five asphalt binder becomes smaller with the increase of temperature. This indicated that five asphalt binder had become closer to viscous fluids in the temperature range. The addition of nanosilica does not change the viscoelastic nature of the asphalt material. It retains a small amount of elastic component at higher temperatures. While OCS-Silica, SOC-Silica and SCA-Silica exerts the advantages of nanosilica, it can form a more stable system with asphalt. With the increase of temperature, the decrease rate of complex shear modulus and the conversion rate of elastic components of hydrophobic nanosilica modified asphalt become slow compared with base asphalt and nanosilica modified asphalt, which proves the recoverable deformation increased in the hydrophobic nanosilica modified asphalt.

The research on permanent deformation of asphalt mixture is generally at 60 °C, but the fact is that the permanent deformation of asphalt mixture will produced within a certain temperature range. Shenoy proposed G*/[1−(sinδtanδ)^−1^] as a refinement to G*·sinδ. The parameter was derived through a semi-empirical approach. It represents the inverse of the non-recoverable compliance and is derived by linking the strain response in the creep experiment with the complex modulus G* from oscillatory shear experiments at a matched timescale. This parameter is more sensitive to phase angle than the Superpave parameter; therefore, it better explains the changes in elastic properties when adding the polymeric modifier. In this paper, the Shenoy rutting parameter was adopted to evaluate the ability of modified asphalt and unmodified asphalt to resist permanent deformation. 

**Figure 12:**
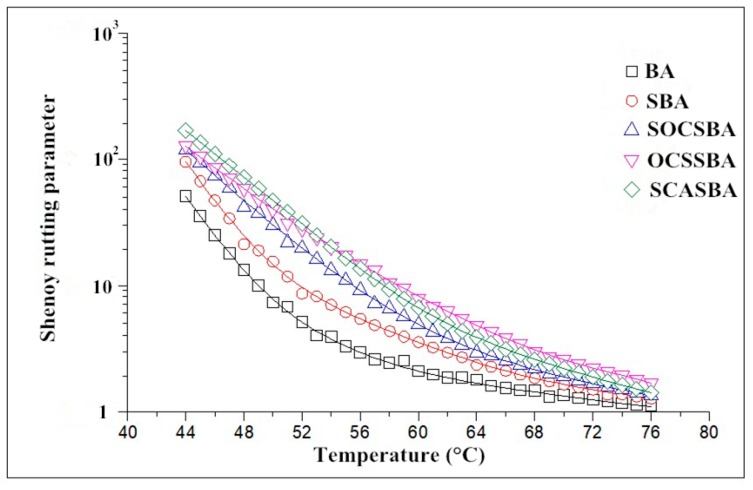
The relationship between Shenoy rutting parameter and temperature for modified and unmodified asphalt.

Figure 12 shows the relationship between Shenoy rutting parameterShenoy rutting parameter and temperature. The changes of the Shenoy rutting parameters of five asphalt binders were relatively consistent with the changes of complex shear mudulus. The Shenoy rutting parameter of SBA has a certain improvement compared with BA, which manifested that the incorporation of nanosilica improves the high temperature performance of asphalt. The Shenoy rutting parameter of OCSSBA, SOCSBA, and SCASBA has a significant improvement compared with SBA, which indicated that incorporation of hydrophobic nanosilica can better improve the high temperature stability and resistance to high temperature permanent deformation of asphalt. 

**Figure 13:**
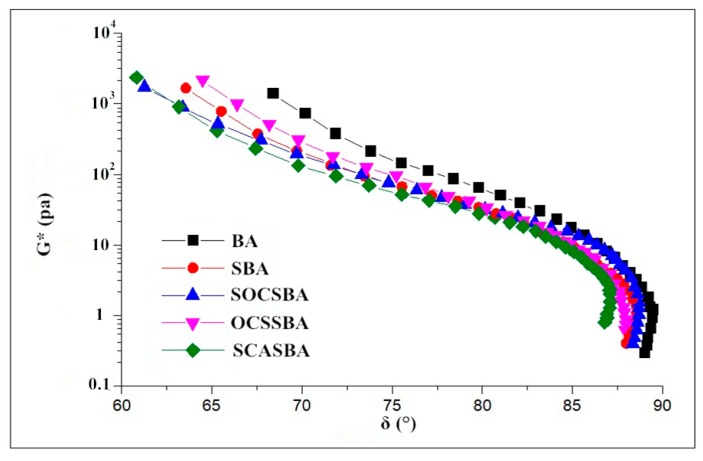
Black diagram for five modified asphalt binders.

Complex shear modulus and phase angle obtained from temperature sweep tests can construct black diagram. Figure 13 shows the viscoelastic data of hydrophobic nanosilica modified asphalt using the black diagram. As shown in Figure 13, the modified asphalt has a smaller phase angle than the base asphalt in the case of the same complex shear modulus. The temperature of pavement in summer is generally around 60 °C, and the complex shear modulus of asphalt is about 5 KPa, the sequence of phase angle for the modified and unmodified asphalt binder were BA > SOCSBA > SBA > OCSSBA > SCASBA, respectively. This indicated that an increase in the elastic behavior and a better rutting resistance for hydrophobic nanosilica modified asphalt. From the curve trend, there is a certain gap between OCSSBA, SOCSBA SCASBA, and SBA, which also reflects the modification mechanism of hydrophobic nanosilica grafted with organic groups in asphalt is different from that of nanosilica.

#### 4.5.2. Frequency Sweep Test Results

The frequency sweep tests of three hydrophobic nanosilica modified asphalt, nanosilica modified asphalt and base asphalt were carried out, and the complex shear modulus (G*), phase angle (δ), storage modulus G*·cosδ with frequency were analyzed.

**Figure 14:**
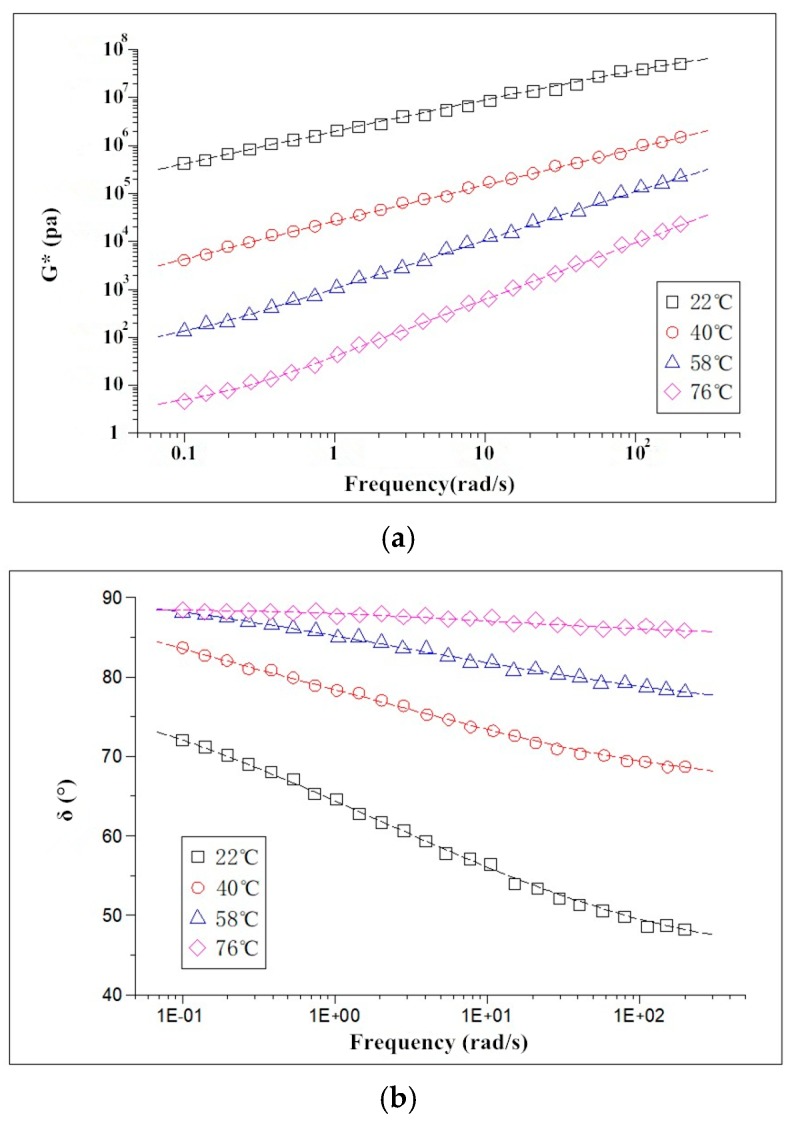
The changes of complex shear modulus and phase angle of base asphalt with frequency. (**a**) complex shear modulus (**b**) phase angle.

The test results showed that the change trend of complex shear modulus of modified asphalt with frequency was consistent with that of base asphalt, which indicated that the addition of modifiers did not change the viscoelastic nature of the asphalt material. Figure 14 shows the change of complex shear modulus and phase angle of BA with frequency at 22 °C, 40 °C, 58 °C, and 76 °C. As can be seen in Figure 14, with the increase of frequency, the phase angle reduced gradually at the same temperature, while the complex shear modulus increased gradually. This indicated that the asphalt exhibited greater elastic properties as the frequency increased, while the phase angle was maintained at a plateau at 76 °C, which may be the reason that asphalt material has been approximated to viscous fluids viscous fluids caused by the increase in temperature, and the effect of load frequency on the elastic composition of the material is also greatly reduced.

**Figure 15:**
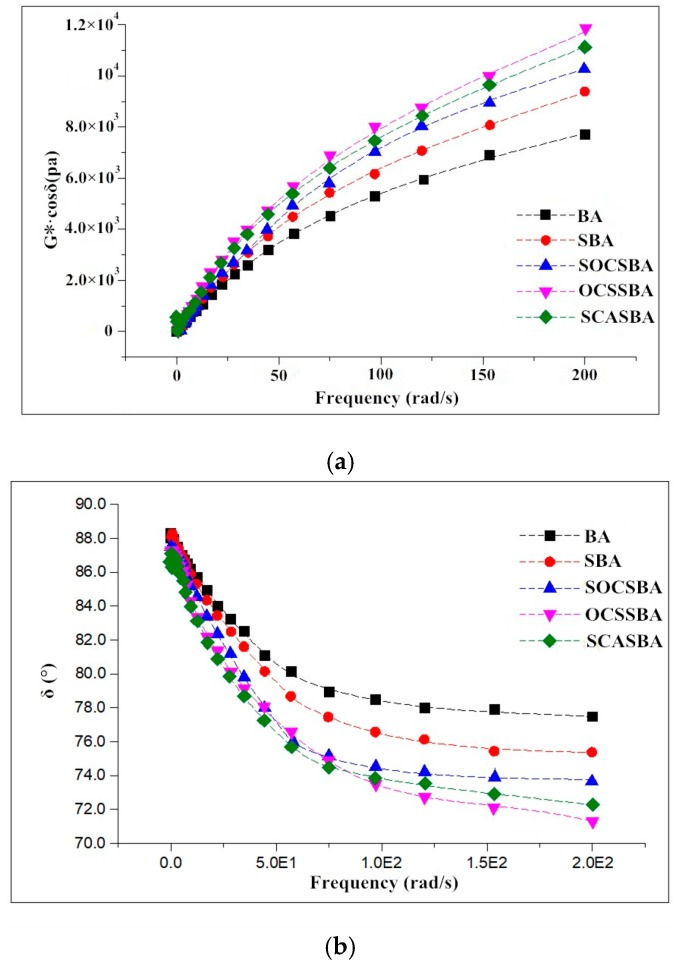
The changes of storage modulus and phase angle of five asphalt binder with frequency at 58 °C. (**a**) storage modulus (**b**) phase angle.

In the summer, the pavement is most likely to produce permanent deformation under continual load and the reference temperature is 58 °C. Figure 15 shows the changes of storage modulus and phase angle of five asphalt binder with frequency at 58 °C. As can be seen in Figure 15, the storage modulus of BA and modified asphalt increased with the increase of the frequency, and the storage modulus of hydrophobic nanosilica has a larger increase than hydrophilic nanosilica. Under the driving load, the vibration frequency of the pavement structure is about 15 Hz (94.2 rad/s). When the load frequency is 100 rad/s, the storage modulus of the BA is 0.508 KPa, and the storage modulus of SBA, SOCSBA, SCASBA, and OCSSBA is 0.589 KPa, 0.683 KPa, 0.745 KPa and 0.712 KPa. The improve of the storage modulus of SBA, SOCSBA, SCASBA and OCSSBA relative to BA is 15.9%, 34.4%, 40.2%, and 46.7%. Thus, the effect of hydrophobic white nanosilica on the storage modulus of asphalt is significant. This is because the nanosilica grafted with organic groups increases the complex shear modulus of asphalt on the one hand, and reduces the phase angle of the asphalt on the other hand, thereby increasing the storage modulus of the asphalt material. This can improve the deformation recovery of the asphalt and reduce the occurrence of permanent deformation during the hydrophobic nanosilica modified asphalt in application. 

As shown in Figure 15, the phase angle of BA and modified asphalt decreased with the increase of the frequency, and the phase angle of five asphalt binders has a large drop before the frequency of 100 rad/s, and then the frequency has less influence on the phase angle of five asphalt binders. At a frequency of 100 rad/s, the phase angle of the hydrophobic nanosilica modified asphalt is reduced by 2–4° compared to hydrophilic nanosilica modified asphalt, which contributes greatly to the storage modulus of the asphalt at high temperatures.

#### 4.5.3. Master Curve Generation

**Figure 16:**
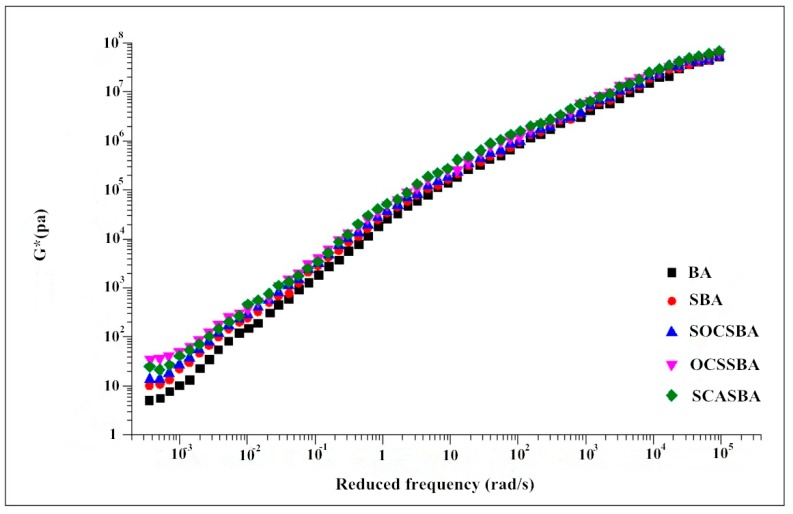
Complex shear modulus master curves for five asphalt binders.

The complex shear modulus of modified asphalt in Figure 16 increased after mixing with modifiers at low and high frequency region compared with base asphalt. In the high frequency region, the complex modulus of five asphalts is relatively close, while in the low frequency region, the complex modulus of five asphalts has a large gap. Moreover, the hydrophobic nanosilica materials modified asphalt exhibits a higher complex modulus than BA and SBA, and the trend of the master curve of the hydrophobic nanosilica materials modified asphalt is relatively moderate, indicating that the hydrophobic nanosilica materials modified asphalt has a relatively low sensitivity and dependence on frequency and exhibits strong stability compared with BA and SBA. 

The reason is that hydrophilic nanosilica acts as a physical cross-linking points in the asphalt. When the load acts on the modified asphalt, it can transmit a certain stress through the hydrophilic nanosilica, thereby the stability of the whole system is improved. However, a large amount of hydroxyl groups exist on the surface of the hydrophilic nanosilica, so that the nanoparticles are in a non-thermodynamic stable state, and the nanoparticles tend to agglomerate, forming a large-sized agglomerate with several weak interfaces, resulting in poor dispersion in asphalt.

The hydrophobic nanosilica successfully grafted with organic groups reduces the formation of agglomerates. In addition to the advantages of hydrophilic nanosilica, the hydrophobic nanosilica has better dispersion in asphalt due to the hydrophobic and oleophilic property. According to the previous analysis, it can be seen that –Si(CH_3_)_3_ on the surface of OCS-Silica, –O–Si–CH=CH_2_ on the surface of SOC-Silica and the –HN_2_, –SH on the surface of SCA-Silica can be more closely combined with the organic components in the asphalt through the coupling effect, so that the hydrophobic nanosilica modified asphalt has more advantage against the load.

#### 4.5.4. Compatibility Analysis Based on Cole–Cole Diagram

**Figure 17:**
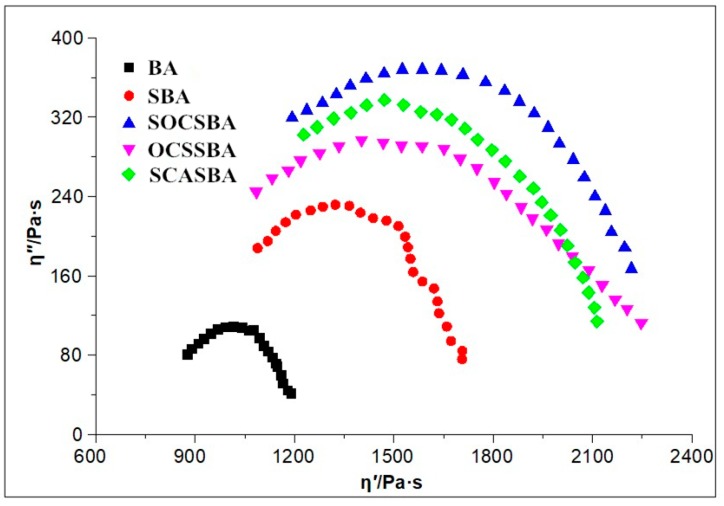
Cole–Cole diagrams of five asphalt binders at 58 °C.

**Figure 18:**
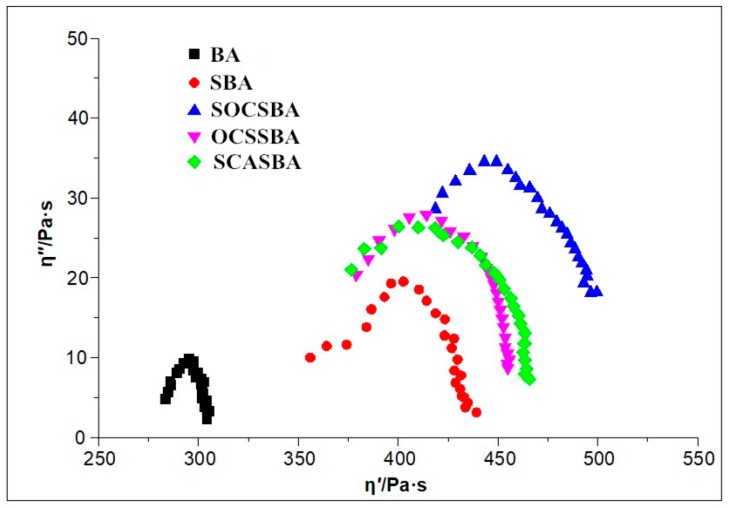
Cole–Cole diagrams of five asphalt binders at 76 °C.

Figure 17 and Figure 18 shows Cole–Cole diagrams of five asphalt binders at 58 °C and 76 °C. 

As can be observed, η” shows its descending following its ascending as the increase of η’, where occurs peak value in the curves. What’s more, the form of Cole–Cole plots differ from each other and base asphalt together with three kinds of hydrophobic nanosilica materials modified asphalt shows the best symmetrical parabolas, followed by nanosilica modifed asphalt (Figure 17). Data concentrated on the left side of the Cole–Cole curves mean the essential elastic properties at the tested temperature,whereas those shifted to the right show a transition from an elastic behavior at low temperature to a viscous behavior as the temperature increases. For all samples studied at 58 °C in this research, the curves occurred in the right side, predicting the dominant viscous behavior. The curves of goodness of fit for parabolas show that the compatibility of asphalt Table 7 shows the regression equation of the Cole–Cole curve of five asphalt binders at 58 °C and 76 °C. The *R*^2^ index represents the correlation coefficient between the Cole–Cole curve and symmetrical parabolas. The higher the R^2^ index, the higher the correlation of Cole–Cole curve and symmetrical parabolas, and the better the compatibility of the modified asphalt. The sequence of R^2^ for the modified and unmodified asphalt binder at 58 °C were BA > SCASBA > OCSSBA > SOCSBA > SBA, respectively. This indicated that the compatibility of SBA is the worst and the surface modified nanosilica exhibits good compatibility with asphalt compared with untreated nanosilica. It is noteworthy that the form of Cole–Cole plots drastically changed and deviated from symmetrical parabolas as the higher temperatures (Figure 17 and Figure 18), which shows the compatibility of asphalt becomes worse as the temperature increases. The sequence of *R*^2^ for the modified and unmodified asphalt binder at 76 °C were BA > SOCSBA > OCSSBA > SCASBA > SBA, respectively.

In summary, the three hydrophobic nanosilica particles modified by different surface modification method have better compatibility with the asphalt compared with unmodifed nanosilica partilces at different temperatures, and form a more uniform blend system with asphalt. 

There are three reasons to explain this phenomenon. First, the surface of the hydrophobic nanosilica particles is not easily affected by moisture to cause agglomeration, and physical mixing can produce more particles of original size. Second, according to the principle of similar compatibility, the three hydrophobic nanosilica particles shows lipophilic property. It is easier to move and homogenize in the organic phase system of asphalt subjected to the high-speed shearing. Third, the connection mechanism between the surface of the modified nanosilica particles and asphalt has changed.

### 4.6. Viscoelastic Analysis of Asphalt Mixture

#### 4.6.1. Static Creep Test Results

**Figure 19:**
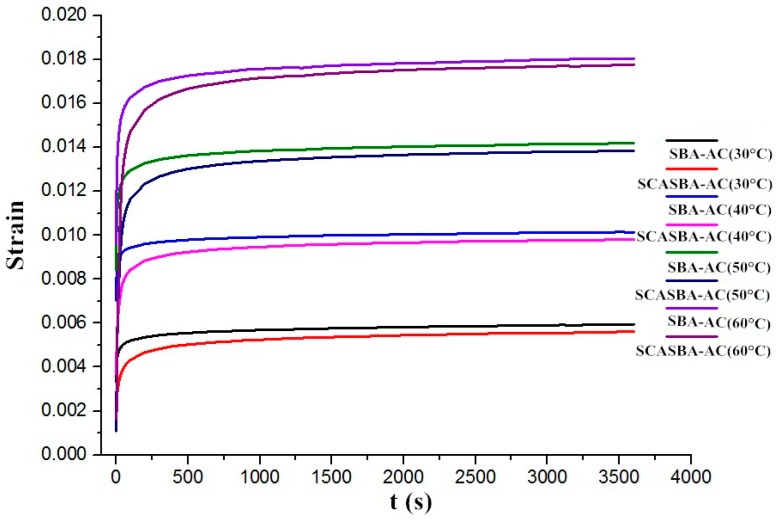
Static creep strain curves of SBA-AC and SCASBA-AC at different temperatures.

The corresponding static creep strain plotted against the loading time is shown in Figure 19. It can be seen that the static creep strain curves of all asphalt mixtures was basically the same, and the deformation can be divided into three parts: the instantaneous elastic part, which is generated at the initial stage of loading, can be regarded as the instantaneous loading of the initial stress; viscoelastic delay part, the deformation growth rate of the asphalt mixture gradually decreases with time; deformation stable part, the deformation tends to a stable growth rate (the creep failure stage is not reached in this experiment). As the temperature increases, the strain of the asphalt mixture increases. At the beginning, the creep strain increment is very large. After 100 s, the creep strain increment gradually slows down. After 500 s, the creep strain increment gradually stabilizes and decreases with the increase of temperature. The addition of SCA-Silica can significantly reduce the creep strain of asphalt mixture at the same temperature, and the reduction descreases as time increases. The creep strain difference of SBA-AC and SCASBA-AC gradually increases with increasing temperature.

In order to further study the improvement of the viscoelasticity of asphalt concrete by adding SCA-Silica, the static creep Burger’s model parameters of SBA-AC and SCASBA-AC based on Levenberg-Marquardt algorithm was calculated separately, as shown in Table 8. It should be taken in cosideration that by now, some more refined model have been developed in the literature. Pasetto et al. introduced a visco-elasto-plastic constitutive model for the characterisation of stress-strain behaviour in bituminous mixtures [35]. Li et al. obtained the Burger’s viscoelastic parameters including E-1, eta (1) E-2, eta (2) and the steady-state creep rate K according to the creep curve, and analyzed the effects of test temperature, stress level and aggregate gradation on the viscoelastic parameters of asphalt mixture [36].

It can be seen from Table 8 that the Burgers model parameters gradually decrease with increasing temperature, and the addition of SCA-Silica increases the elastic modulus (E_1_) of asphalt mixture, and the elastic modulus (E_2_) and viscosity coefficient (η_1_, η_2_) are correspondingly reduced. In addition, E_2_ belongs to the Kelvin models, and its variation is affected by the parallel viscosity coefficient (η_2_). Thus, it can be considered that, in a certain temperature range, the addition of SCA-Silica reduces the viscosity and enhances the elasticity of the asphalt mixture, so that the instantaneous elastic deformation is reduced, the viscous flow deformation is increased, and the delayed deformation of the viscoelasticity is increased.

#### 4.6.2. Dynamic Creep Test Results

**Figure 20:**
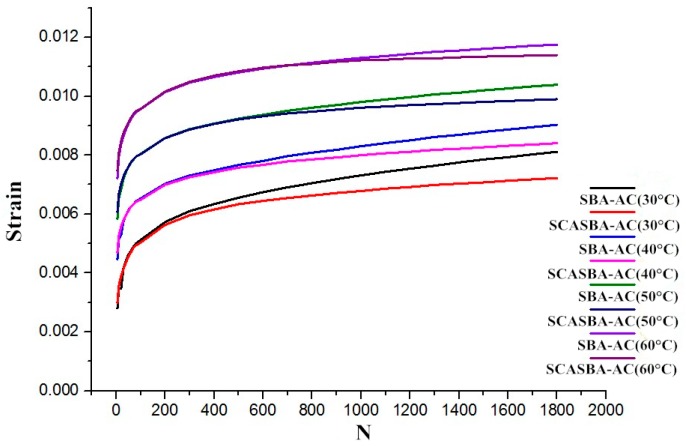
Dynamic creep strain curves of SBA-AC and SCASBA-AC at different temperatures.

The characteristic change in dynamic creep strain can be interpreted as follows: as the number of cycles increases, the dynamic creep strain increases. It is interesting to note that the dynamic creep strain tends to increase with an increasing number of cycles only during the first 200 cycles; thereafter, the dynamic creep strain increment becomes negligibly small (Figure 20). Progressive increment in dynamic creep strain is obvious for all test specimens. The addition of SCA-Silica can reduce the creep strain of asphalt mixture at the same temperature, but it is not obvious at the beginning. When the number of cycles is the same, the reduction gradually decreases with the increase oftemperature. At the same temperature, as the number of cycles increases, the difference of SBA-AC and SCASBA-AC gradually increases.

In order to further study the improvement of the viscoelasticity of asphalt concrete by adding SCA-Silica, the dynamic creep Burgers model parameters of SBA-AC and SCASBA-AC based on Levenberg-Marquardt algorithm was calculated separately, as shown in Table 9.

It can be seen from the above table that with the increase of temperature, the viscosity coefficient (η_1_) of asphalt mixture increases under dynamic loading, and the elastic modulus (E_2_) and viscosity coefficient (η_2_) decrease gradually. When static load is applied, the incorporation of SCA-Silica increases the elastic modulus (E_1_) of the asphalt mixture, the elastic modulus (E_2_) and viscosity coefficient (η_1_, η_2_) decrease correspondingly, so that the instantaneous elastic deformation is reduced, the viscous flow deformation and the delayed deformation of the viscoelasticity is synchronously increased. When dynamic load is applied, the incorporation of SCA-Silica makes the elastic modulus (E_2_) and viscosity coefficient (η_2_) of asphalt mixture gradually decrease, the viscosity coefficient (η_1_) increases, the viscous flow deformation decreases, and the delayed deformation of the viscoelasticity increases.

Overall, the hydrophobic nanosilica modifier can effectively improve the viscoelasticity property of asphalt and asphalt mixture. The price of normal asphalt is about 3000 rmb/t, and the price of hydrophobic nanosilica modified asphalt is about 3300 rmb/t. The comprehensive unit price analysis of mechanical paving hydrophobic nanosilica modified asphalt concrete of 7 cm thick is as follows. (a) Artificial cost: 2.1 rmb/cm^2^ (b) Materials cost (including main material and auxiliary material): 97.7 rmb/cm^2^ (c) Mechanical cost: 2.5 rmb/cm^2^ (d) Other cost (including safe and civilized construction costs, fees and taxes): 7.4 rmb/cm^2^. The total cost of hydrophobic nanosilica modified asphalt concrete is 109.7 rmb/cm^2^, and the total cost of normal asphalt concrete is 102 rmb/cm^2^. The cost of mechanical paving hydrophobic nanosilica modified asphalt concrete of 7 cm thick is 7.6% higher than normal asphalt concrete. Considering the ability of hydrophobic nanosilica modified asphalt to improve the viscoelasticity property of the asphalt concrete, an increase of 7.6% of the cost is still acceptable.

## 5. Conclusions

In this paper, the effect of hydrophobic nanosilica on the viscoelasticity property of asphalt and asphalt mixture was systematically investigated according to conventional physical properties test, SEM test, FTIR test, DSC test, DSR test, static creep test, and dynamic creep test. The main conclusions are as follows: 

The conventional physical properties test results illustrated that addition of hydrophobic nanosilica results in relatively harder binder compared to hydrophilic nanosilica, which may be beneficial for rutting resistance. Moreover, the addition of three hydrophobic nanosilica materials could reduce the temperature sensibility of asphalt, and improve the viscosity and anti-aging property.

1. The SEM test, FTIR test, and DSC test were carried to explore the modification mechanism of hydrophobic nanosilica. The test results showed that the hydrophobic nanosilica had stronger adhesion property and can be better distributed in the asphalt without agglomeration. In addition, newly formed chemical bonds were found in the hydrophobic nanosilica modified and its mixture with stone, which is the biggest difference from the modification mechanism of hydrophilic nanosilica modified asphalt.
2. Temperature sweep test and frequency sweep test results showed that hydrophobic nanosilica modified asphalt had a lower sensitivity and dependence on temperature and frequency than hydrophilic nanosilica modified asphalt, showing strong stability. The Cole–Cole diagrams indicated that the compatibility of SBA was the worst and the surface modified nanosilica exhibited good compatibility with asphalt compared with untreated nanosilica.
3. Through static and dynamic creep test, it found that the addition of hydrophobic nanosilica can significantly reduce the creep strain at the same temperature. When static load is applied, the reduction descreases as time increases, and the creep strain difference of SBA-AC and SCASBA-AC gradually increases with increasing temperature. When dynamic load is applied, the reduction gradually decreases with the increase of temeprature. At the same temperature, as the number of cycles increases, the difference of SBA-AC and SCASBA-AC gradually increases.

## Figures and Tables

**Table 1:** Technical parameters of asphalt.

Technical Parameters	Penetration			25 °C Ductility	Softening Point	Wax Content	Flash Point	Solubility	Density
	15 °C	25 °C	30 °C						
Units	0.1 mm			cm	°C	%	°C	%	g·cm^−3^
Test results	22.8	67.3	109.8	>130	48.8	1.8	328	≥99.9	1.036
Test procedure	GB/T0606-2011			GB/T0605-2011	GB/T0606-2011	GB/T0615-2011	GB/T0611-2011	GB/T0607-2011	GB/T0603-2011

**Table 2:** Technical parameters of four nanosilica materials.

Type	Water Characteristics	BET (m^2^/g)	Average Particle Size (nm)	Loss on Drying (105 °C, 2 h, wt %)	Loss on Ignition (1000 °C, 2 h, wt%)	pH Value	SiO_2_ Content (wt %)
Silica	Hydrophil-ia	130 ± 25	12	≤1.5	≤1	3.7–4.7	≥99.8
OCS-Silica	Hydropho-bicity	110 ± 20	16	≤0.5	≤2	3.6–4.3	≥99.8
SOC-Silica	Hydropho-bicity	100 ± 25	14	≤0.5	≤2	4.0–6.0	≥99.8
SCA-Silica	Hydropho-bicity	125 ± 20	12	≤0.5	1.5–2.5	5.0–8.0	≥99.8
Standard values	-	130 ± 30	≤20	≤3.0	≤10.0	3.7–6.5	≥99.8
Test procedure	GB/T 20020	GB/T 20020	GB/T 20020	GB/T 20020	GB/T 20020	GB/T 20020	GB/T 20020

**Table 3:** The gradation of AC-16.

Sieve (mm)	16	13.2	9.5	4.75	2.36	1.18	0.6	0.3	0.15	0.075
Gradation (wt %)	95	88	73	46	31	21.5	15.5	11.5	5.5	6

**Table 4:** The conventional physical properties tests results of BA, SBA, OCSSBA, SOCSBA, and SCASBA.

Property	BA	SBA	OCSSBA	SOCSBA	SCASBA
15 °C Penetration (d-mm)	22.8	18.4	26.7	32.5	23.5
25 °C Penetration (d-mm)	67.3	56.7	64.1	66.1	58.9
30 °C Penetration (d-mm)	109.8	92.7	93	100.5	88.5
PI	−0.87	−1.06	0.64	1.45	0.24
Softening Point (°C)	48.8	50.1	54.4	53.7	53.7
10 °C Ductility (cm)	45.7	21.5	25.5	20.6	17.6

**Table 5:** The viscosity test results of BA, SBA, OCSSBA, SOCSBA, and SCASBA.

Property	BA	SBA	OCSSBA	SOCSBA	SCASBA
Rotational Viscosity at 115 °C (pa.s)	1.397	2.195	2.673	6.786	3.531
Rotational Viscosity at 135 °C (pa.s)	0.4503	0.7724	1.125	2.978	1.987
Rotational Viscosity at 155 °C (pa.s)	0.1927	0.2621	0.512	1.483	0.684

**Table 6:** Physical parameter of BA, SBA, OCSSBA, SOCSBA, and SCASBA before and after rolling thin film oven (RTFO) test.

Materials	Unaged Binder			RTFO Aged Binder		
	Weight (g)	25 °C Penetration (d-mm)	10 °C Ductility (cm)	Weight (g)	25 °C Penetration (d-mm)	10 °C Ductility (cm)
BA	35.21	67.3	45.7	35.0	42.6	12.2
SBA	35.52	56.7	21.5	35.4	38.54	9.2
OCSSBA	35.41	64.1	25.5	35.3	47.49	14.9
SOCSBA	35.25	66.1	20.6	35.2	44.58	13.7
SCASBA	35.31	58.9	17.0	35.2	40.74	8.6

**Table 7:** Regression equation of the Cole-Cole curve of five asphalt binders at 58 °C and 76 °C.

Temperature	Materials	Regression Equation	*R* ^2^
58 °C	BA	$y=-0.0016x^{2}+3.1951x-1466.5$	0.9945
	SBA	$y=-0.001x^{2}+2.617x-1483.7$	0.9756
	OCSSBA	$y=-0.0003x^{2}+0.896x-361.66$	0.9904
	SCASBA	$y=-0.0005x^{2}+1.5916x-848.81$	0.9924
	SOCSBA	$y=-0.0004x^{2}+1.3948x-712.94$	0.9818
76 °C	BA	$y=-0.0138x^{2}+7.9311x-1133$	0.9702
	SBA	$y=-0.0051x^{2}+4.0859x-793.78$	0.8339
	OCSSBA	$y=-0.0024x^{2}+1.9758x-380.09$	0.9323
	SCASBA	$y=-0.0048x^{2}+3.9745x-798$	0.8874
	SOCSBA	$y=-0.0052x^{2}+4.6083x-990.86$	0.9506

**Table 8:** Static creep Burger’s model parameters of asphalt mixture under different temperatures.

Parameters	SBA-AC 30 °C	SCASBA-AC 30 °C	SBA-AC 40 °C	SCASBA-AC 40 °C	SBA-AC 50 °C	SCASBA-AC 50 °C	SBA-AC 60 °C	SCASBA-AC 60 °C
E_1_	36.5	56.0	19.7	31.8	15.7	18.7	13.3	16.7
E_2_	114.1	62.7	83.6	35.0	44.3	15.1	28.3	10.4
η_1_	627,332.3	566,977.3	453,161.0	346,585.7	329,777.6	203,886.5	225,465.8	163,449.7
η_2_	3632.8	3386.6	1271.1	959.2	860.7	356.4	525.9	273.0
R	0.973	0.987	0.982	0.991	0.975	0.991	0.979	0.994

**Table 9:** Dynamic creep Burger’s model parameters of asphalt mixture under different temperatures.

Parameters	SBA-AC 30 °C	SCASBA-AC 30 °C	SBA-AC 40 °C	SCASBA-AC 40 °C	SBA-AC 50 °C	SCASBA-AC 50 °C	SBA-AC 60 °C	SCASBA-AC 60 °C
E_2_	15.5	15.1	12.3	12.2	8.3	8.3	4.8	4.7
η_1_	75,557.3	97,184.9	85,349.1	114,612.4	90,641.0	137,603.3	134,588.0	168,913.1
η_2_	333.7	251.3	121.2	107.8	64.5	59.6	50.3	42.4
R	0.973	0.987	0.982	0.991	0.975	0.991	0.993	0.993

## References

[1] Yuan J., Wang J.Y., Xiao F.P., Amirkhanian S., Wang J., Xu Z.Z. (2017). Impacts of multiple-polymer components on high temperature performance characteristics of airfield modified binders. Constr. Build. Mater..

[2] Özen H., Aksoy A., Tayfur S., Çelik F. (2008). Laboratory performance comparison of the elastomer-modified asphalt mixtures. Build. Environ..

[3] Pasetto M., Baldo N. (2017). Unified approach to fatigue study of high performance recycled asphalt concretes. Mater. Struct..

[4] Ahmedzade P., Gunay T., Grigoryeva O., Starostenko O. (2017). Irradiated recycled high density polyethylene usage as a modifier for bitumen. J. Mater. Civ. Eng..

[5] He B.Y., Yu J.Y., Gu Y., Zhuang R.H., Sun Y.B. (2018). Rheological properties of lignosulfonate intercalated layered double hydroxides modified bitumen before and after ultraviolet aging. Constr. Build. Mater..

[6] Liu K.F., Zhang K., Shi X.M. (2018). Performance evaluation and modification mechanism analysis of asphalt binders modified by graphene oxide. Constr. Build. Mater..

[7] Gao J.F., Wang H.N., You Z.P., Hasan M.R.M., Lei Y., Irfan M. (2018). Rheological behavior and sensitivity of wood-derived bio-oil modified asphalt binders. Appl. Sci..

[8] Sahebzamani H., Alavi M.Z., Farzaneh O. (2018). Evaluating effectiveness of polymerized pellets mix additives on improving asphalt mix properties. Constr. Build. Mater..

[9] Zhao Z.J., Xu S., Wu W.F., Yu J.Y., Wu S.P. (2015). The aging resistance of asphalt containing a compound of LDHs and antioxidant. Petrol. Sci. Technol..

[10] Kheradmand B., Muniandy R., Hua L.T., Solouki A. (2015). A Laboratory investigation on the rheological properties of aged and unaged organic wax modified asphalt binders. Petrol. Sci. Technol..

[11] Liu J.Y., Li P. (2012). Low temperature performance of sasobit-modified warm-mix asphalt. J. Mater. Civ. Eng..

[12] Woszuk A., Panek R., Madej J., Zofka A., Franus W. (2018). Mesoporous silica material MCM-41: Novel additive for warm mix asphalts. Constr. Build. Mater..

[13] Golewski G.L. (2017). Generalized fracture toughness and compressive strength of sustainable concrete including low calcium fly ash. Materials.

[14] Woszuk A. (2018). Application of fly ash derived zeolites in warm-mix asphalt technology. Materials.

[15] Zhang Y., Leng Z., Zou F.L., Wang L., Chen S.S., Tsang D.C.W. (2018). Synthesis of zeolite A using sewage sludge ash for application in warm mix asphalt. J. Cleaner Prod..

[16] Feldman D. (2014). Polymer nanocomposites in building, construction. J. Macromol. Sci. A Pure Appl. Chem..

[17] Fang C.Q., Yu R.E., Liu S.L., Li Y. (2013). Nanomaterials applied in asphalt modification: A review. J. Mater.Sci. Technol..

[18] Taherkhani H., Afroozi S., Javanmard S. (2017). Comparative study of the effects of nanosilica and zyco-soil nanomaterials on the properties of asphalt concrete. J. Mater. Civ. Eng..

[19] Saltan M., Terzi S., Karahancer S. (2017). Examination of hot mix asphalt and binder performance modified with nano silica. Constr. Build. Mater..

[20] Firouzinia M., Shafabakhsh G. (2018). Investigation of the effect of nano-silica on thermal sensitivity of HMA using artificial neural network. Constr. Build. Mater..

[21] Jiangkongkho P., Arksornnukit M., Takahashi H. (2018). The synthesis, modification, and application of nanosilica in polymethyl methacrylate denture base. Dent. Mater. J..

[22] Zhang L., Zhang D.F. (2005). Study on the surface modification and characterization of nano-SiO_2_. Russ. J. Inorg. Chem..

[23] Ekin N., Icduygu M.G., Gultek A. (2016). Investigation of long-term water absorption behavior of carbon fabric reinforced epoxy composites containing hydrophobic nanosilica. Turk. J. Chem..

[24] Suriyaprabha R., Karunakaran G., Kavitha K., Yuvakkumar R., Rajendran V., Kannan N. (2014). Application of silica nanoparticles in maize to enhance fungal resistance. IET Nanobiotechnol..

[25] Wang Q., Zhang Q., Huang Y.H., Fu Q., Duan X.J., Wang Y.L. (2008). Preparation of high-temperature vulcanized silicone rubber of excellent mechanical and optical properties using hydrophobic nano silica sol as reinforcement. Chin. J. Polym. Sci..

[26] Ani Q.F., Lyu Z.J., Shangguan W.C., Qiao B.L., Qin P.W. (2018). The synthesis and morphology of a perfluoroalkyl oligosiloxane@SiO_2_ resin and its performance in anti-fingerprint coating. Coatings.

[27] Milionis A., Languasco J., Loth E., Bayer I.S. (2015). Analysis of wear abrasion resistance of superhydrophobic acrylonitrile butadiene styrene rubber (ABS) nanocomposites. Chem. Eng. J..

[28] Fini E.H., Hajikarimi P., Rahi M., Nejad F.M. (2016). Physiochemical, rheological, and oxidative aging characteristics of asphalt binder in the presence of mesoporous silica nanoparticles. J. Mater. Civ. Eng..

[29] Rasool R.T., Song P., Wang S.F. (2018). Thermal analysis on the interactions among asphalt modified with SBS and different degraded tire rubber. Constr. Build. Mater..

[30] Liu K.F., Zhang K., Wu J.L., Muhunthan B., Shi X.M. (2018). Evaluation of mechanical performance and modification mechanism of asphalt modified with graphene oxide and warm mix additives. J. Cleaner Prod..

[31] Shenoy A. (2002). Model-fitting the master curves of the dynamic shear rheometer data to extract a rut-controlling term for asphalt pavements. J. Test. Eval..

[32] Xue Y., Wu S., Hou H., Zha J. (2006). Experimental investigation of basic oxygen furnace slag used as aggregate in asphalt mixture. J. Hazard. Mater..

[33] Ahmedzade P., Sengoz B. (2009). Evaluation of steel slag coarse aggregate in hot mix asphalt concrete. J. Hazard. Mater..

[34] Pasetto M., Baldo N. Comparative performance analysis of bituminous mixtures with EAF steel slags: A laboratory evaluation. Proceedings of the 2008 global symposium on recycling, waste treatment and clean technology, REWAS 2008.

[35] Pasetto M., Baldo N. (2015). Computational analysis of the creep behaviour of bituminous mixtures. Constr. Build. Mater..

[36] Li P., Jiang X., Guo K., Xue Y., Dong H. (2018). Analysis of viscoelastic response and creep deformation mechanism of asphalt mixture. Constr. Build. Mater..

